# The Correlations Between Concussions and Chronic Traumatic Encephalopathy (CTE) in the National Football League: Why Does Society Keep Promoting Sports With High CTE Rates?

**DOI:** 10.7759/cureus.64359

**Published:** 2024-07-11

**Authors:** Mazen Zamzam, Kenan Sinan, Hashem Mohilldean, Sazid Hasan, Ehab S Saleh

**Affiliations:** 1 Orthopedic Surgery, Oakland University William Beaumont School of Medicine, Rochester, USA; 2 Neurology, Wayne State School of Medicine, Detroit, USA; 3 Orthopedic Surgery, University of Toledo Medical Center, Toledo, USA; 4 Orthopedic Surgery, Corewell Health, Rochester, USA

**Keywords:** orthopedic surgery, sport injury, helmet use, national football league, sports-related concussion, concussion

## Abstract

The National Football League (NFL) is a highly popular sport in the United States, attracting numerous aspiring athletes due to its lucrative pay and fame. However, the pursuit of a career in the NFL comes with significant health risks, particularly concussions and their long-term effects. Repeated head traumas in the NFL can lead to chronic traumatic encephalopathy (CTE), a neurodegenerative disease that is characterized by a spectrum ranging from cognitive and behavioral aberrations and has been linked to conditions such as Parkinson's and Alzheimer's diseases. Despite growing evidence, NFL officials have historically downplayed the connection between concussions and CTE, attributing symptoms to other factors such as performance-enhancing drugs. To address the concussion crisis, the NFL has implemented rule changes and partnered with engineers to develop safer helmets. However, the most effective approach to combating CTE involves early detection through MRI brain scans, which are a potential method for identifying the disease in living patients and subsequently facilitating early intervention. While other contact sports such as boxing have been shown to increase the risk of traumatic brain injury as well as CTE, the impact the NFL has on CTE is the most prominent in today's society. This editorial emphasizes the need for the NFL to acknowledge the clear link between concussions and CTE and to invest in comprehensive diagnostic and therapeutic strategies such as new monoclonal antibody therapies. Despite ethical and technical challenges, such as the use of embryonic stem cells and the risks associated with radioactive scans, advancing these methods could save lives and improve the long-term health outcomes of current and former NFL players. Enhanced understanding and proactive management of CTE are crucial for mitigating the severe impact of concussions in professional football.

## Editorial

The National Football League (NFL) is one of the most widely observed sports in the United States. It is also a competitive field enticed by players who far exceed the capacity of available positions. The NFL is so highly coveted by aspiring athletes due to the lucrative paychecks earned by players and the fame that comes with it. Although the profitable payouts and fame may seem appealing, the cost of obtaining the money and fame comes at a much higher price than players first realize. In an article published in the National Library of Medicine, 368 total concussions were reported missing at least one game due to concussion over four NFL seasons between 2018 and 2022 [1]. The concussions suffered are only a gateway to the various potential diseases that may arise from repeated blows to the head. Abundant hits to the head cause chronic traumatic encephalopathy (CTE), which researchers have discovered in former NFL players, as seen in a study published in the Journal of Legal Medicine [2]. The diagnosis of CTE can only be done post-mortem by identifying the buildup of a protein called tau. The protein affects neurological functions of the brain and is linked to other neurological diseases such as Parkinson's and Alzheimer's [3]. Although there is clear evidence of the link between concussions and CTE in the NFL, it is still continuously denied by many.

NFL Commissioner Roger Goodell and NFL team owner Jerry Jones are two of the chief disputants regarding the link between CTE and concussions. In an article released by the National Institute of Health, even though there is clear evidence that shows the detrimental effects of trauma on the brain, the NFL did not address this issue until 2009 [4].

Although numerous post-mortem examinations confirm CTE in former NFL players, the NFL asserts that the findings of CTE result not from the repeated concussions suffered by players but rather from the use of performance-enhancing drugs, as mentioned in Miller’s article "A Late Hit for Pro Football Players” that was published on Science [4]. In an article released by the American Orthopedic Society for Sports Medicine, Mack et al. bring to light data collected between 2015 and 2019 and identified a total of 1,302 concussions of which 80% occurred in NFL games [5]. In the results, he concluded that, due to the concussion reduction strategies employed by the NFL in 2018, there was a sustained two-year decrease in concussion incidence.

To address the growing concern about head injuries to football players, the NFL is collaborating with engineers to create helmets that will minimize the impact of head injuries. In an article published by Frontiers in Bioengineering and Biomedical, football elements using finite element (FE) helmets consisting of 21 liquid shock absorbers were developed. The results of this helmet model showed promising results to improve head safety and reduce the risk of brain injury by demonstrating a nearly 33% decrease in head impact compared to normal helmets. Even though the NFL has taken several initiatives in collaboration with other companies, head injuries are an inevitable part of playing in the NFL, and the most effective technique would be to actively perform MRI brain scans on current and former NFL players to detect CTE. Assuming these claims, the purpose of this research argument is to offer clear evidence of the link between CTE and concussions and offer insight into possible solutions regarding CTE in living patients.

In the Journal of Legal Medicine, Drysdale [2] offers insight into why the NFL should scan current and former players for CTE, as head injuries are inevitable when playing in the NFL. Drysdale refers to two former players' autopsies regarding the discovery of a high concentration of tau in their brains, a protein that indicates the diagnosis of CTE [2]. Drysdale also mentions a lawsuit filed against the NFL by former players, who claimed that the NFL played a significant role in the risk of head injuries suffered by players, and there was no initiative taken to prevent the risk of head injuries [2]. Drysdale considers the rule changes implemented by the NFL, including shortening kickoffs, banishing hits to the neck and above, and suspending players for violating any enforced rules. The author states these points to convey his main argument. Regardless of the rule changes implemented or settlements of lawsuits, head injuries are an inevitable part of the NFL, and the most effectivetechnique would be to scan current and former NFL players to detect CTE actively. In the journal article "A Late Hit for Pro Football Players," Miller discusses former professional wrestler Chris Nowinski, who had a short-lived career due to several concussions that left him prone to memory lapses, irritability, and sleeplessness [4]. Nowinski pursued a different career and took a job at a life sciences consulting firm, hoping to return to wrestling someday. However, after discovering the long-term effects of head injuries in athletes, he was left uneasy. Nowinski co-founded a nonprofit organization that promotes research on sports-related head injuries. Nowinski had convinced over 130 football players and other athletes to donate their brains for research when they passed away [4]. Boston University Medical School and independent researchers claim to have discovered signs of pathology of CTE in a total of 12 former players in the NFL [5]. Nowinksi held a press conference at a Super Bowl in 2009 and announced the discovery of pathology in an 18-year-old high school football player [4]. Ann Mckee, a neuropathologist at Boston University, discovered a high concentration of tau protein in a deceased person's brain, which is a prime suspect in several neurodegenerative disorders such as Alzheimer's. However, the tissue used to discover the accumulation of tau did not come from an Alzheimer's patient but rather from a former NFL player, John Grimsely, who died at age 45 [4]. After further investigation, researchers concluded that Grimsely's pathology fits the diagnosis of CTE. Bennett Omalu was the first to document CTE in a football player. However, after his research and discoveries, he was acknowledged with an overwhelming amount of skepticism [4]. Much of the skepticism came from the NFL, who claimed that the finding was due to performance-enhancing drugs and not the nature of the game of football. While there have only been case reports regarding this connection, there have yet to be any clinical trials or controlled trials demonstrating this relationship. Exactly how repetitive hits to the brain may cause delayed neurodegeneration is still unknown, but the claims made by various researchers are not claims to be ignored [4,5]. In an article released by the National Library of Medicine, the authors aim to address the rising concern of concussions in sports and the unknown long-term consequences, affecting participants at all levels. This study reveals traumatic head injuries not only have an immediate impact, such as concussions, on the health of NFL players, but also significant and detrimental long-term neural and cognitive outcomes [1]. Repetitive head trauma stands implicated in the pathogenesis of CTE, a progressive neurodegenerative disorder characterized by a spectrum of cognitive and behavioral aberrations including dementia, memory impairment, impulsivity, confusion, and affective disturbances. An investigation by Mack et al. in 2017 underscored the pervasive nature of this affliction within the NFL community, reporting neuropathological evidence indicative of CTE in a staggering 99% of posthumously examined brains sourced from former players. Due to the increased awareness of the long-term consequences, the NFL began to implement changes to the rules of the game and protective equipment to improve the safety of the players [5]. The authors conclude that, with the implementation of the "targeting" rule and changes to kickoff rules following the 2017-2018 NFL season, there was a notable decline in the total number of concussions per year among NFL players. Additionally, the average number of games missed per concussion decreased, indicating a positive trend in concussion management. While both offensive and defensive units saw reductions in concussion rates and severity, variations based on player positions persisted. Notably, offensive unit players experienced less reduction in concussion rates compared to defensive unit players. Defensive backs and tight ends emerged as particularly vulnerable positions, with a higher risk of sustaining concussions resulting in missed games. Although efforts have been made to safeguard players, the findings underscore the ongoing challenge of managing concussions in professional football. The updated insights into concussion epidemiology should inform targeted interventions by the NFL aimed at minimizing risks, especially for players in high-risk positions.

In an article released by Annals of Biomedical Engineering, helmet design and safety evaluation are increasingly incorporating considerations of brain strain, recognized as a key factor in concussion. This study examines the potential impact of various helmet designs on brain strain, assessing two widely utilized metrics: peak maximum principal strain (MPS) across the entire brain and cumulative strain damage measure (CSDM). It is essential for the NFL to recognize the consequences of head injuries and collaborate with companies to minimize head injuries due to the long-lasting impact on the NFL players. As discussed in the article released by the National Institute of Health, the liquid helmet model demonstrated superior performance in terms of Head Injury Assessment Risk Metric (HARM), exhibiting the lowest values across 33 out of 36 impact scenarios. It achieved an average reduction of 33.0% and 32.0% compared to existing helmet models in subconcussive and concussive tests. Additionally, the liquid helmet attained a helmet performance score of 0.71, contrasting with scores ranging from 1.07 to 1.21 observed in the other four finite element (FE) helmet models. Moreover, brain strains were consistently lower when using the liquid helmet. This study showcases the potential of liquid shock absorbers to enhance helmet safety, advocating for the creation of physical helmet prototypes integrating this technology [3-5].

Concussions in the NFL are an inevitable part of playing in the league. There have been several attempts at preventing concussions, but all have been proven unsuccessful. The large amount of concussions suffered by players across the league raises concerns for CTE. The most effective way to seek any solution to CTE is first to recognize the clear link between the concussion culture in the NFL and CTE. Once the NFL recognizes that CTE is a severe concern playing NFL, various initiatives can be funded to first detect CTE in living patients and then eventually devise a possible cure for the disease. Researchers at the North Shore Neurological Institute detected signs of CTE in living patients using low radioactivity brain scans. However, no complete diagnosis has been made. Devising a brain scan using the brain scan utilized by the researchers at the North Shore Neurological Institute as a template will be the most effective way to discover possible solutions to the disease. However, devising a brain scan that is safe enough to detect CTE through the skull may not be a quick enough solution to a current and demanding problem. In another approach, an experiment can be conducted using current and former NFL players as both the experimental group and the control group. CTE. impairs neurological functions of the brain, which may lead to memory loss, impaired judgment, dementia, and several other symptoms. Supposing these claims, a player first entering the NFL will undergo tests that challenge their neuronal functions, such as their judgment and memory. Using these results, after a player has played through several years in the NFL, tests can be retaken and compared to their initial results, and a possible diagnosis can be made based on the most recent results. Once a diagnosis has been successfully confirmed in living patients, possible cures can be more effectively researched and potentially discovered.

CTE is discovered post-mortem by examining the brain for lost tissue, which is replaced by tau [5]. A possible solution to the disease of CTE may be through the use of immunotherapy. As stated in an article in the National Institute of Health, therapeutic monoclonal antibody emerges as a leading approach to combat CTE. Given that tau accumulation stands out as a primary characteristic of CTE, it becomes a compelling target for antibody-based therapeutic interventions. Although a solution to the threatening disease of CTE is immediately necessary for members of the NFL discourse community, there are several limitations. The NFL must first acknowledge the clear link between the concussions suffered by its current players and the post-mortem diagnosis of CTE in its former players. The researchers at North Shore Neurological Institute were not successfully able to fully diagnose a patient with CTE using a low radioactivity brain scan. Devising a brain scan that will be able to diagnose CTE in living patients entirely comes at the risk of using a more significant amount of radioactivity, which may lead to threats of cancer in the patient. The practice of stem cell regeneration is a very controversial topic as the stem cells come from the embryo. To obtain the cells from an embryo, it must be aborted, and its chance at life will be terminated.

However, barring these limitations, the methods proposed to diagnose and cure CTE successfully may be applied immediately to current and former players. The methods may also be extended to further understand the disease's effects and perhaps quicker and more efficient solutions than the use of stem cells from an embryo. The NFL has seen several self-inflicted mortalities, who were later diagnosed with CTE. Implementing the proposed methods may enhance the knowledge of certified experts regarding CTE and ultimately save the lives of current and former NFL players.

The NFL stands as a highly coveted yet perilous arena, where the allure of lucrative paychecks and fame masks the grave risks associated with repetitive head trauma. The substantial incidence of concussions among NFL players, as evidenced by numerous studies, underscores the pervasive and severe nature of these injuries. CTE, linked to repeated concussions, presents a serious long-term health threat to players, manifesting in a range of debilitating neurological symptoms. Despite clear scientific evidence, the NFL has historically denied the connection between concussions and CTE, delaying crucial safety reforms. Recent efforts, including advancements in helmet technology and rule changes, aim to mitigate head injuries, but these measures fall short of eliminating the risks inherent in the sport. Moving forward, it is imperative for the NFL to acknowledge the concussion-CTE link, invest in diagnostic innovations for early detection of CTE in living players, and explore potential therapeutic interventions. Only through a concerted and transparent approach can the NFL hope to safeguard the health and futures of its athletes, addressing the dire consequences of head injuries and fostering a safer playing environment.
